# Evaluation of Carotenoid Bioaccessibility in Selected Dietary Sources Using a Modified INFOGEST *In Vitro* Digestion Protocol

**DOI:** 10.1021/acsfoodscitech.6c00187

**Published:** 2026-09-09

**Authors:** Elena Rodríguez-Rodríguez, Begoña Olmedilla-Alonso, Rocío Estévez-Santiago, Milagros Sánchez-Prieto

**Affiliations:** ‡ Department of Chemistry in Pharmaceutical Sciences, Analytical Chemistry, VALORNUT Group (920030-UCM), Pharmacy School, Universidad Complutense de Madrid (UCM), Avenida Complutense, 28040 Madrid, Spain; § Department of Metabolism and Nutrition, Institute of Food Science, Technology and Nutrition (ICTAN-CSIC), C/José Antonio Novais 6, 28040 Madrid, Spain

**Keywords:** carotenoids, lutein, zeaxanthin, bioaccessibility, *in vitro* digestion, INFOGEST, food matrix, mixed micelles

## Abstract

This study assessed the bioaccessibility and stability of lutein and other carotenoids in avocado, kiwi, orange, and lamb’s lettuce. A modified INFOGEST *in vitro* digestion protocol was employed to improve analytical recovery, micellar separation, and quantification of carotenoids. The standardized static *in vitro* digestion model from COST Action INFOGEST was adapted by increasing the sample size to 20 g and applying centrifugation at 20000*g* for 5 min to enhance micellar transfer. Carotenoids were quantified using high-performance liquid chromatography. Lamb’s lettuce exhibited the highest lutein (4615 μg/100 g) and β-carotene (2156 μg/100 g) contents and showed low recovery and poor β-carotene bioaccessibility (5.1%). Lutein in avocado (172 μg/100 g) showed high recovery (95.9%) and bioaccessibility (55.3%). Kiwi (65 μg/100 g lutein) demonstrated the highest lutein bioaccessibility (71.0%) and good stability (73–80% recovery), with β-carotene bioaccessibility at 36.4%. Orange was rich in β-cryptoxanthin (73 μg/100 g) and zeaxanthin (13 μg/100 g) and exhibited moderate bioaccessibility for violaxanthin (25.3%), β-cryptoxanthin (18.0%), and zeaxanthin (30%). These findings highlight the importance of sample mass and centrifugation parameters in assessing carotenoid bioaccessibility. The results highlight how food matrix and carotenoid type affect bioaccessibility, reinforcing the need to adapt digestion protocols to lipophilic compound properties and the food matrix.

## Graphical abstract

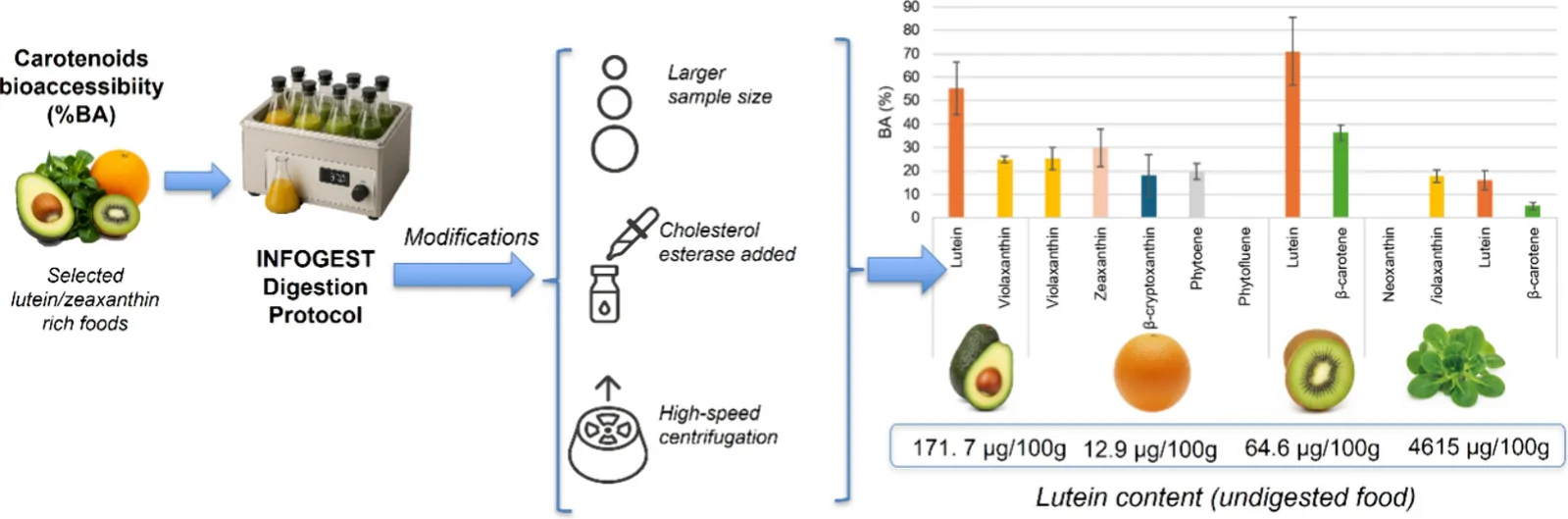

## 1 Introduction

Carotenoids are natural, fat-soluble pigments synthesized by plants, algae, fungi, archaea, and certain bacteria. They are recognized for their various biological activities. These compounds have a wide variety of molecular structures, giving them antioxidant and pro-vitamin properties (some, such as β-carotene, are precursors of vitamin A), as well as important roles in vision and immune health.^1^ Lutein and zeaxanthin, dietary xanthophylls with well-established roles in ocular health, particularly in protecting the macula from oxidative damage and age-related degeneration, have been associated with increased levels in blood and macular pigment optical density and improved visual function following dietary intake.^1–4^ However, their effective bioavailability remains limited, in part due to low bioaccessibility (BA), which refers to the fraction of carotenoids released from the food matrix and incorporated into mixed micelles during digestion, a necessary step prior to intestinal absorption.^1,2^

Human absorption of carotenoids, especially from raw foods, is generally inefficient due to physical barriers in plant tissues, competition among lipophilic compounds, and limited solubilization. Therefore, measuring BA *in vitro* is critical for designing interventions that enhance carotenoid intake.^1,5^

The INFOGEST protocol has been widely adopted as a standardized model to simulate human gastrointestinal digestion *in vitro* studies.^6^ However, several publications have indicated that this protocol may require specific adaptations when applied to lipophilic compounds such as carotenoids, due to their low solubility in aqueous environments and the limited hydrolysis efficiency of their esterified forms.^5,7^ In this regard, several studies have successfully adapted the protocol to analyze carotenoids, with modifications typically involving additional steps to account for their lipophilic nature and the need for efficient micellarization and extraction. Specifically, adaptations often include enhanced micelle separation and carotenoid extraction, where after the digestion phases, methods are incorporated to efficiently separate the micellar fraction containing the solubilized carotenoids, commonly involving centrifugation at high speeds to isolate the aqueous phase with mixed micelles, followed by exhaustive extraction with organic solvents to recover the carotenoids for quantification.^5,7^ Furthermore, adaptation of enzyme concentrations or activities may be applied. For example, inclusion of specific gastric lipases may be considered as an effective adaptation to the standard INFOGEST protocol, since although it outlines the basic enzymatic setup, the addition of enzymes such as rabbit gastric extract can significantly enhance the lipolysis of plant-based food matrixes.^8^ These adaptations collectively aim to provide a more accurate estimation of carotenoid BA, overcoming the inherent limitations of the general INFOGEST protocol for these important lipophilic compounds.

Therefore, in the present study, methodological modifications in the original INFOGEST protocol^6^ were introduced to facilitate carotenoid recovery and micellar phase separation for reliable quantification, while maintaining the structure of the INFOGEST static model. These included using a larger sample size (20 g instead of the standard 5 g), the incorporation of cholesterol esterase in the intestinal phase to facilitate xanthophyll ester hydrolysis,^9^ the assay of different centrifugation methods to remove the undigested sample and to conduct experiments with different solvents (diethyl ether: hexane: dichloromethane and diethyl ether: methanol: methyl *tert*-butyl ether) to carry out the extraction of carotenoids in the digested food.

The aim of this study was to evaluate the BA of lutein and other carotenoids in select fruits (avocado, kiwi, and orange) and leafy green vegetables (lamb’s lettuce) using an adapted INFOGEST *in vitro* digestion procedure.^6^ A preliminary comparison between two digestion approaches was conducted only to identify the most suitable conditions for carotenoid release and micellarization; the adapted protocol was then applied to all food matrixes.

## 2 Materials and Methods

### 2.1 Reagents

The following reagents were used to prepare the simulated digestion fluids: KCl (Merck, Darmstadt, Germany), MgCl_2_(H_2_O)_6_, and (NH_4_)_2_CO_3_ (Sigma-Aldrich, St. Louis, MO, USA) and KH_2_PO_4_, NaHCO_3_, NaCl, HCl, and CaCl_2_(H_2_O)_2_ (Panreac, Barcelona, Spain).

Standard digestive enzymes namely α-amylase from porcine pancreas (EC 3.2.1.1; ref A3176; ≥ 10 U/mg solid), pepsin from porcine gastric mucosa (EC 3.4.23.1; ref P6887; 3706 U/mg protein), cholesterol esterase from porcine pancreas (EC 3.1.1.13; ref C3766; ≥50 U/mg protein), bile from bovine and ovine (EC 232-369-0; ref B8631), and pancreatin from porcine pancreas (EC 232-468-9; ref P1750) were purchased from Sigma-Aldrich (St. Louis, MO, USA).

Acetone, *tert*-butyl methyl ether (tBME), methanol (MetOH), ethanol, hexane, dichloromethane (DCM), petroleum ether (PE), and diethyl ether (DE) were obtained from Análisis Vínicos (Tomelloso, Spain). Lutein (xanthophyll from marigold), zeaxanthin, α- and β-carotene, β-cryptoxanthin, violaxanthin, neoxanthin, phytoene, phytofluene, tocopherol acetate, potassium hydroxide (KOH), pyrogallic acid, and trimethylamine were obtained from Sigma-Aldrich (Madrid, Spain).

### 2.2 Samples

Avocados (Hass), kiwi (Oscar, variety Hayward), oranges (Nave-Late), and lamb’s lettuces (Machuca, in prepackaged 150g trays) were purchased from Mercamadrid in Madrid, Spain, in March 2017. The selected foods were chosen due to their high or representative lutein content and their habitual consumption in Spain. This study forms part of a broader research project on carotenoid bioavailability;^10^ however, the present manuscript focuses exclusively on the *in vitro* digestion experiments. From these, 3 pieces of each food (2 trays in the case of lamb’s lettuce) were saved for analysis (both fresh and after undergoing the digestion process). Each fruit piece weighed approximately 150–250 g depending on the food type, while each lamb’s lettuce tray contained 150 g. All food samples were stored under refrigeration (4 °C) and protected from light immediately after purchase. Analytical procedures were performed within a maximum of 3 days to minimize carotenoid degradation and ensure sample integrity.

### 2.3 Extraction, Saponification, and High-Performance Liquid Chromatography (HPLC) Analysis of Carotenoids

The fruits were manually peeled, the seeds were removed, and the edible portion was homogenized in a hand blender for sufficient time to obtain a puree. The lamb’s lettuce was homogenized directly using the same method. Appropriate sample amounts were allocated either for carotenoid analysis (10–20 g per replicate, depending on color intensity) or for use in the *in vitro* digestion assay (20 g per sample). To carotenoids analysis in nondigested foods, each sample was prepared in triplicate, with each replicate ranging from 10 to 20 g based on its color intensity. The homogenized fruit puree was placed in a mortar and ground with Celite, together with 3.5 mL of α-tocopheryl acetate (0.32 mg/mL) as internal standard and 20 mL of acetone (w/v ratio ranging from 1:1 to 1:2). The solid residue, initially colored, was re-extracted twice with fresh acetone under the same conditions until colorless. With each extraction, the acetone became increasingly colored as carotenoids were solubilized, while the residue progressively lost its pigmentation. The combined acetone extracts were transferred to a separatory funnel containing a 1:1 (v/v) mixture of DE:PE. Distilled water and 10% NaCl solution were added, and the mixture was vigorously shaken and allowed to separate. The upper organic phase containing carotenoids was collected, and residual moisture was removed by addition of anhydrous sodium sulfate. The purified sample was then evaporated to dryness using a rotary evaporator. For analysis, the dry residue was redissolved in an injection solvent consisting of tBME and MetOH (50:50) and passed through a 0.45 μm filter to remove particulates.^11,12^

To hydrolyze carotenoid esters and clear the carotenoid extract of interfering substances, such as chlorophylls and unwanted lipids, the extraction of carotenoids (from raw foods) was followed by alkaline saponification of the extract according to the procedure of Granado et al. (2001)^13^ with slight modifications.^12^ For analysis, aliquots from each food extract (400 μL) were saponified. The process involved mixing the aliquot with 0.1 M pyrogallic acid in ethanol and 30% KOH in MetOH, followed by 7 min of ultrasonication. The extract was then washed with water, followed by extraction with a 1:1 DE/PE (50:50) mixture, using vortexing and centrifugation. Finally, the supernatant was dried under nitrogen, reconstituted in MetOH/tBME (50:50), and prepared for HPLC injection.

Carotenoid concentrations were determined by HPLC using a system consisting of a model 600 pump, a Rheodyne injector and a 2998 diode array detector (DAD) (Waters, Milford, MA, USA), and a C30 YMC column (5 μm, 250 × 4.6 mm i.d.) (Waters, Wilmington, MA, USA) with a guard column (Aquapore ODS type RP-18) at room temperature (25 °C). Mobile phase was formed by MetOH with 0.1% trimethylamine (solvent A) and tBME (solvent B) in a linear gradient. At baseline, 25, 55, and 60 min the ratios of the solvents were 95:5, 70:30, 35:65, and 95:5. The detection was performed at 450 nm for carotenoids phytoene at 285 nm and phytofluene at 370 and 285 nm for tocopherol acetate (internal standard). Chromatograms were processed using Empower 2 software (Waters, Milford, MA, USA). Quantification was performed using standard curves.

The concentrations of the carotenoids in the curve were 0.75–3.75 ng/μL for lutein (*R* ^2^ = 0.999), 0.6–3 ng/μL for zeaxanthin (*R* ^2^ = 0.999), 1.04–5.25 ng/μL for β-cryptoxanthin (*R* ^2^ = 0.997), 0.45–2.25 ng/μL for α-carotene (*R* ^2^ = 0.998), 1.05–4.2 ng/μL for β-carotene (*R* ^2^ = 0.980), and 1.15–11.4 ng/μL for violaxanthin (*R* ^2^ = 0.989). The recoveries of the internal standard analyzed were between 85.8% and 117.6%. The precision was evaluated by the relative coefficient of variation (% CV) which ranges from 4.23 (β-carotene) to 4.79 (lutein). LOD values ranged between 0.008 and 0.040 μg/mL, and LOQ values between 0.025 and 0.120 μg/mL, depending on the carotenoid.

All carotenoid concentrations reported in this study are expressed on a fresh weight basis of the edible portion.

### 2.4 Selection of Digestion Protocol and the Extraction Method for Carotenoids from Digested Foods

Two different protocols of *in vitro* digestion were tested using fresh oranges (*Citrus sinensis* L.) (Figure 1) before the *in vitro* digestion of the foods selected for the study. Both were based on the standardized static *in vitro* digestion model proposed by the COST action INFOGEST^6^ plus cholesterol esterase (3077 U/mL). This enzyme was included to enhance the hydrolysis of esterified carotenoids, particularly xanthophylls such as lutein and zeaxanthin, which are poorly cleaved under standard INFOGEST conditions. Previous studies have demonstrated that while carotenoids and tocopherols remain stable during digestion, the hydrolysis of xanthophyll esters specifically requires cholesterol esterase, as human pancreatic lipase alone is insufficient for this purpose,^9^ thereby supporting its inclusion in the protocol. The digestion process included oral, gastric, and small intestinal phases, followed by the separation of the supernatant. For both protocols, the oral phase involved 3.5 mL of oral fluid, 25 μL of CaCl_2_, and 0.5 mL of an α-amylase solution (1500 U/mL) at 37 °C for 2 min. The gastric phase involved 7.5 mL of gastric fluid, 5 μL of CaCl_2_, and 1.6 mL of a pepsin solution (25000 U/mL), with the pH adjusted to 3 with HCl at 37 °C. The small intestine phase included 10 mL of intestinal fluid, 40 μL of CaCl_2_, 5 mL of a pancreatin solution (800 U/mL, trypsin activity) and 2.5 mL of bile salts solution (160 mM), and 1 mL of a cholesterol esterase solution (3077 U/mL), with the pH adjusted to 7 with NaOH at 37 °C. Pancreatin stock activity (800 U/mL) corresponds to trypsin units, following INFOGEST recommendations and manufacturer specifications. Upon completion each phase, samples were placed in an ice bath. In accordance with the INFOGEST protocol, enzymes should ideally be incorporated based on their experimentally determined activity to ensure physiological relevance and reproducibility. However, in our study, enzymatic activities were not directly measured; instead, we relied on the declared activity values provided by the manufacturer, which were used as reference to calculate the final activities (U/mL) in each digestion phase.

**Figure 1:**
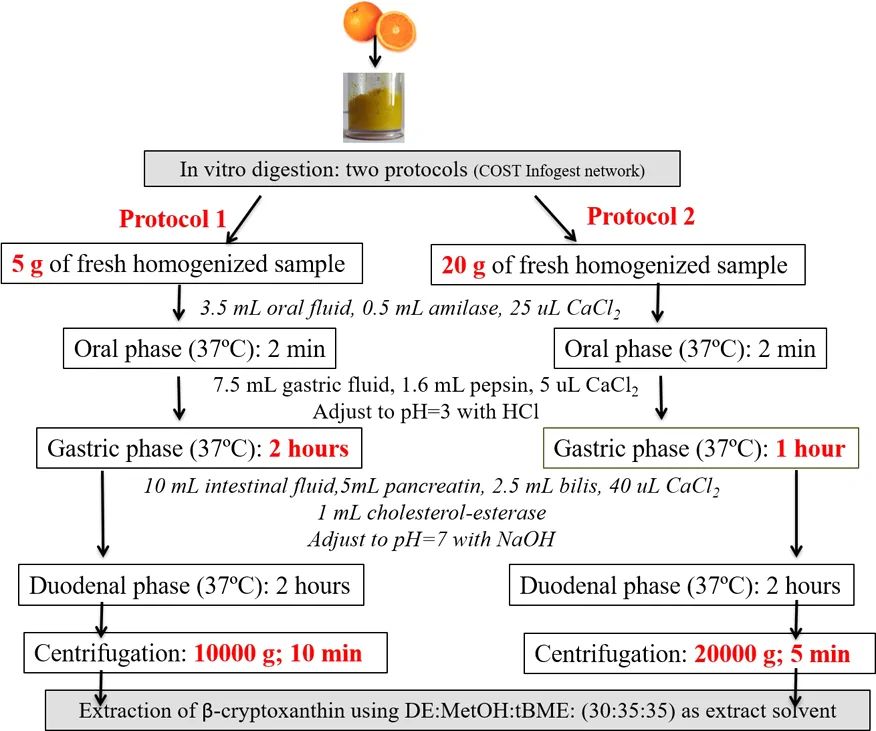
Scheme of two different *in vitro* digestion protocols tested to assess BA of β-cryptoxanthin from orange (*Citrus sinensis* L.). Note: In Protocol 2, the enzyme concentrations were quadrupled proportionally to the increased sample weight (20 vs 5 g) to maintain equivalent enzymatic activity per gram of food.

This represents a methodological limitation, as relying on manufacturer-declared activities may introduce variability and reduce physiological precision. Although INFOGEST emphasizes the importance of activity-based dosing, several published studies applying the protocol to lipophilic micronutrients have used manufacturer-declared activities when experimental assays were not technically feasible or when enzymes lack standardized activity units.^14,15^ Our study followed this widely adopted practical approach, but we recognize that future work should incorporate experimental determination of enzymatic activities to enhance physiological relevance and reduce batch-to-batch variability.

It is important to clarify that increasing the sample mass from 5 to 20 g did not involve modifying the volumes of SSF, SGF, or SIF, which remained identical to those specified in the INFOGEST protocol. What was increased 4-fold was the amount of enzyme added, to maintain the same enzyme to substrate ratio. The stock enzyme solutions were not more concentrated; instead, proportionally larger volumes of the same stock solutions were used. The final activities in the digestion mixture were approximately 188 U/mL α amylase in the oral phase, 4400 U/mL pepsin in the gastric phase, 216 U/mL trypsin activity (from pancreatin), 166 U/mL cholesterol esterase, and 21.6 mM bile salts in the intestinal phase. These details ensure full reproducibility of the modified protocol.

This approach has been adopted in previous applications of the INFOGEST method, particularly when technical constraints prevent direct measurement. Given that enzymes were sourced from reputable suppliers with standardized quality controls, this method is considered acceptable within the framework of the protocol and supports reproducibility under practical laboratory conditions.

In accordance with the INFOGEST protocol, gastric lipase was not included in the digestion model. The use of gastric lipase is considered optional in INFOGEST due to its limited commercial availability, the lack of standardized activity units, and the resulting challenges for interlaboratory reproducibility. For these reasons, many studies applying INFOGEST protocol, particularly those focused on lipophilic micronutrients, omit gastric lipase and rely on the intestinal phase as the main contributor to lipolysis.^9,16,17^ Regarding enzyme activities, pancreatin was added following the standardized mass-based recommendations of INFOGEST, which are derived from manufacturer-reported proteolytic and lipolytic activities and ensure consistent enzyme-to-substrate ratios across studies. Although enzymatic activities were not experimentally determined in this work, the use of these standardized equivalents provides reproducible digestion conditions and aligns with common practice in carotenoid BA research.

- Protocol 1: A total of 5 g of homogenized sample was mixed with reagents specified by INFOGEST^6^ plus cholesterol esterase (3077 U/mL). The phases lasted 2 min (oral), 2 h (gastric), and 2 h (intestinal phase). After digestion, centrifugation was performed at 10000*g* for 10 min, which were the conditions we followed in previous studies.^12^
- Protocol 2: Similar to Protocol 1 but a 20 g sample was used, and as a result, the enzyme concentration was also quadrupled (aimed to achieve higher absorbance values and reduce analytical determination errors). Furthermore, the gastric phase was shortened to 1 h, and centrifugation was performed at 20000*g* for 5 min (to transfer carotenoids from the intestinal digesta to the aqueous-micellar phase). The centrifugation conditions were selected in based on different assays previously conducted on kiwis (*Actinidia deliciosa*) that were extracted, digested following Protocol 1 and centrifuged at the last step following different conditions: 5000*g*, 20 min; 10000*g*, 5 min; 10000*g*, 10 min; 10000*g*, 20 min; 20000*g*, 5 min. Centrifugation assays conducted on kiwis (Actinidia deliciosa) showed the following BA percentages: 8.9% (5000*g*, 20 min), 10% (10000*g*, 5 min), 6.4% (10000*g*, 10 min), 5.1% (10000*g*, 20 min), and 16.6% (20000*g*, 5 min). These methodological adjustments were introduced to improve analytical detectability and micellar phase clarity, not to increase carotenoid BA beyond physiologically plausible value.

Preliminary trials were conducted to adapt key parameters of the digestion protocol, including sample weight and centrifugation conditions. Increasing the sample weight from 5 to 20 g improved phase separation and carotenoid recovery, particularly in matrixes with low pigment concentration and high-water content. Centrifugation speed and duration were also adjusted to enhance micellar phase clarity and minimize losses. These modifications were empirically selected based on consistent improvements in extract quality and chromatographic signal intensity, although no formal statistical comparison was performed. The adapted conditions were subsequently applied to all food samples.

In this study, the results are interpreted strictly in terms of *in vitro* BA as assessed under standardized INFOGEST conditions. The static digestion model does not aim to reproduce or predict *in vivo* absorption, but rather to provide a controlled and reproducible framework for comparing the relative release and micellarization of carotenoids across different food matrixes. Therefore, the findings should be understood as reflecting differences in *in vitro* BA rather than approximations of physiological uptake.

To extract carotenoids from the digested food fraction, a mixture of DE/MetOH/tBME (30:35:35) was used because it yielded the best results after extracting a digested kiwi sample following Protocol 2. Following this digestion, carotenoids were extracted using (a) DE/hexane/DCM (30:35:35) [using a hexane/DCM mixture (1:5 v/v) because this is what we use for carotenoid extraction in human serum^18^] and (b) DE/MetOH/tBME (30:35:35) (as MetOH and tBME are the mobile phases used in HPLC analysis). In both cases, DE was used as an intermediate polarity solvent. Briefly, the digesta fraction was mixed with the selected solvent mixture for extraction and stirred for 2 min on a stirring plate. Subsequently, after adding 10% NaCl, the mixture was centrifuged (10000*g*, 10 min), and the organic phase was collected. The process was repeated until the aqueous phase was colorless. After combining the extracted organic phases, the solvent was removed using a rotary evaporator, and the residue was reconstituted in MetOH/tBME (50:50). After extraction, the extract was saponified prior to chromatographic analysis, following the previously explained protocol. As already mentioned, solvent mixture b [DE/MetOH/tBME (30:35:35)] offered the best results and was therefore used to extract carotenoids from the remaining digested food samples.

Using the β-cryptoxanthin results obtained from the digested orange extract analyzed following each of the previously mentioned protocols, percentage of BA was calculated according to eq 1 as the ratio between the concentration of carotenoid in the micellar aqueous phase (supernatant) and its respective concentration in the fruit. It represents the fraction of the carotenoid released from the food matrix and incorporated into the micellar (soluble) phase, making it potentially available for absorption.

(carotenoidscontentin themicellaraqueousphase/carotenoidscontentinundigestedfood)×100 1

Under Protocol 1, BA was 1.1% for β-cryptoxanthin, 1.8% for β-carotene, and 0.87% for α-carotene. When applying Protocol 2, which incorporated a larger sample mass and higher-speed centrifugation, BA increased markedly to 47.1% for β-cryptoxanthin, 17.2% for β-carotene, and 8.4% for α-carotene. These results confirm that Protocol 2 consistently improved carotenoid micellarization in orange and justified its selection as the adapted digestion procedure applied to all food matrixes in the study.

It is important to clarify that the comparison between Protocol 1 and Protocol 2 was not designed as a comparative study between digestion models. Instead, this preliminary test using orange was performed solely as a selected step to identify the most suitable conditions for carotenoid micellarization and recovery. Once Protocol 2 showed clearly superior and more consistent performance, it was selected as the best adapted method and applied to all subsequent food samples. Therefore, the aim of the study was not to compare protocols, but to evaluate carotenoid BA across different matrixes under a single adapted digestion procedure.

It should be acknowledged that orange, the matrix used in the preliminary comparison between the two digestion approaches, contains carotenoids predominantly in esterified form. This may have favored the detection of differences between protocols, particularly due to the inclusion of cholesterol esterase. The preliminary test was not intended to compare matrixes or evaluate protocol performance across different carotenoid profiles, but solely to identify practical conditions that improved micellar phase clarity and analytical recovery. It cannot be ruled out that, had matrixes containing mainly free carotenoids been used instead, the differences observed between the two approaches might have been smaller. This limitation has been considered when interpreting the results, which are strictly based on a single adapted INFOGEST protocol applied uniformly to all foods

### 2.5 Digestion of Foods

We adapted a standardized static *in vitro* digestion model, originally proposed for food by the INFOGEST COST action in a consensus paper,^6^ to specifically evaluate carotenoid BA. Thus, the previously named “Protocol 2” was followed to perform the digestion of the selected foods. Initially, eight 20 g samples of each food (which had been homogenized using a blender) were prepared to begin the digestion process. At each stage, two samples were removed to analyze the carotenoid content without centrifugation (digesta, a mixture of liquids, enzymes, and partially digested food) and to study the stability of carotenoids (and any possible alterations these compounds might undergo during *in vitro* digestion). Extraction of the carotenoids was performed using DE/MetOH/tBME (30:35:35), and analysis was done by HPLC. This yielded two samples from the oral phase, two from the gastric phase, two from the intestinal phase, and two that underwent complete digestion (mixed micelles). These last two samples were subjected to selected centrifugation conditions determined through tests with kiwi (20000*g* for 5 min).

## 3 Results

Before presenting the results, it should be noted that only the adapted protocol (Protocol 2) was used for the analysis of all food matrixes, following its selection during the preliminary adaptation step.

### 3.1 Carotenoids Content in Fruits and Vegetables

The analysis of the undigested carotenoid content across various fruits and vegetables revealed distinct profiles for each (Figure 2 and Table 1).

**Figure 2:**
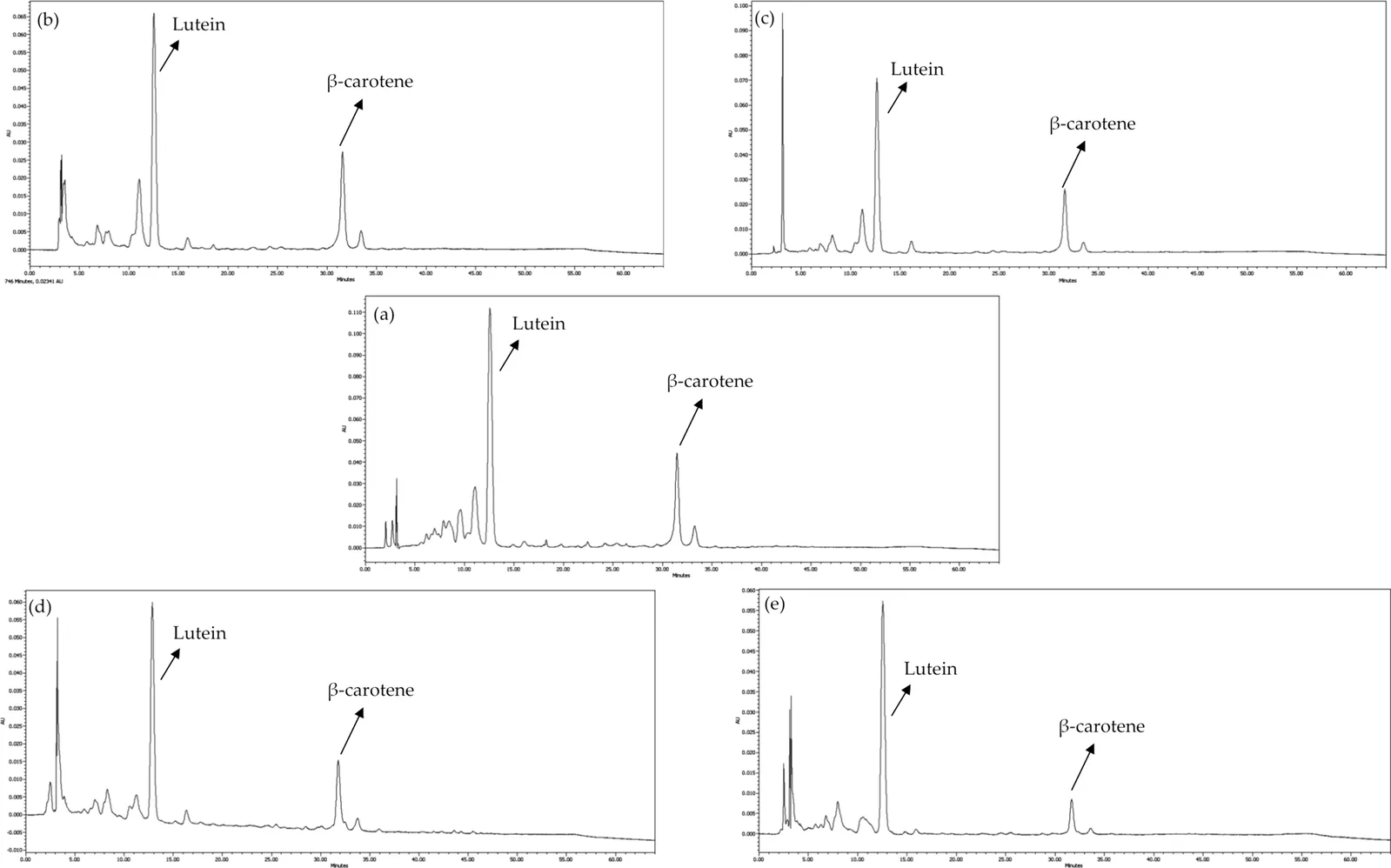
Chromatograms recorded at 450 nm of lutein and β-carotene in kiwi pulp and digested materials after each phase of *in vitro* digestion: (a) undigested kiwi; (b) oral phase; (c) gastric phase; (d) intestinal phase; (e): mixed micelles. All of the chromatograms are on the same scale.

**Table 1:** Carotenoids Content (μg/100 g) in the Selected Pulps Fruits and Lamb’s Lettuce and Digested Materials after Each Phase of *In Vitro* Digestion^a^

undigested food		O	O + G	O + G + I	mixed micelles
Avocado					
lutein	171.7 ± 14.7	143 ± 16.7	53 ± 18.0	164 ± 6.6	95.0 ± 19.2
violaxanthin	128 ± 6.2	41 ± 2.5	n.d.	n.d.	32.0 ± 1.8
Orange					
violaxanthin	51 ± 3.2	45 ± 5.0	19 ± 1.3	18 ± 0.3	13 ± 2.5
zeaxanthin	13 ± 3.2	16 ± 2.7	16 ± 2.4	11 ± 3.4	4 ± 1.9
β-cryptoxanthin	73 ± 7.1	54 ± 19.8	54 ± 7.9	37 ± 10.1	13 ± 6.7
phytoene	7 ± 1.9	2 ± 0.3	2 ± 0.2	2 ± 0.5	1 ± 0.3
phytofluene	Tr	n.d.	n.d.	n.d.	n.d.
Kiwi					
lutein	65 ± 10.6	52 ± 8.4	51 ± 11.2	48 ± 6.0	46 ± 9.3
β-carotene	36 ± 8.5	33 ± 4.2	30 ± 2.0	23 ± 4.2	13 ± 1.2
Lamb’s Lettuce					
neoxanthin	940 ± 261.1	316 ± 64.4	n.d.	n.d.	n.d.
violaxanthin	1536 ± 431.4	302 ± 58.1	n.d.	301 ± 47.1	27 ± 76.2
lutein	4615 ± 618.6	1247 ± 98.5	557 ± 176.6	627 ± 171.9	744 ± 184.6
β-carotene	2156 ± 417.3	698 ± 52.8	682 ± 103.3	348 ± 84.6	110 ± 31.4

a O: oral phase. G: gastric phase. I: intestinal phase. Tr: traces. n.d.: not detectable.

Avocado was found to be a notable source of both lutein, with 171 μg/100 g, and violaxanthin, at 128 μg/100 g. Kiwi presented a moderate carotenoid profile, with lutein (64 μg/100 g) being more abundant than β-carotene (35 μg/100 g). Orange, while containing a broader spectrum of carotenoids, showed its highest concentration in β-cryptoxanthin (73 μg/100 g) and violaxanthin (51 μg/100 g), with zeaxanthin and phytoene present in smaller quantities (12 μg/100 g and 7 μg/100 g respectively). Finally, lamb’s lettuce stood out as exceptionally rich in lutein (4615 μg/100 g) and β-carotene (2156 μg/100 g). It also contained substantial amounts of violaxanthin (1535 μg/100 g) and neoxanthin (940 μg/100 g), indicating a diverse and high carotenoid content.

### 3.2 Stability and BA of Carotenoids after the Digestion Process

The stability and BA of the most abundant carotenoids found in the foods are shown in Table 2 and Figure 3. The recovery (%) of each main carotenoid in the digesta fraction provides information about the stability of the carotenoids during *in vitro* gastrointestinal digestion.

**Table 2:** Recovery of Carotenoids in the Noncentrifuged Digesta and Bioaccessible Content in the Micellar Fraction after *In Vitro* Digestion in the Selected Pulps Fruits and Lamb’s Lettuce

	recovery (%)^a^			bioaccessible content (μg/100 g)
	O	O + G	O + G + I	mixed micelles
Avocado				
lutein	83.4 ± 9.7	31.1 ± 10.5	95.8 ± 3.9	95.0 ± 8.1
violaxanthin	32.1 ± 1.9	n.d.	n.d.	31.9 ± 1.5
Orange				
violaxanthin	87.8 ± 9.8	37.9 ± 2.5	35.1 ± 0.6	5.9 ± 0.4
zeaxanthin	124.4 ± 21.4	125.3 ± 18.6	84.4 ± 26.1	0.1 ± 0.0
β-cryptoxanthin	74.0 ± 27.0	73.4 ± 10.8	50.4 ± 13.8	22.0 ± 2.1
phytoene	26.5 ± 4.6	25.2 ± 2.5	29.5 ± 6.7	1.3 ± 0.3
phytofluene	n.d.	n.d.	n.d.	n.d.
Kiwi				
lutein	80.3 ± 13.0	78.3 ± 17.3	73.7 ± 9.4	45.8 ± 7.5
β-carotene	92.7 ± 11.7	85.7 ± 5.5	63.4 ± 11.7	12.9 ± 3.1
Lamb’s Lettuce				
neoxanthin	33.6 ± 6.9	n.d.	n.d.	n.d.
violaxanthin	19.7 ± 3.8	n.d.	19.6 ± 3.1	271.21 ± 76.23
lutein	27.0 ± 2.1	12.1 ± 3.8	13.6 ± 3.7	744.00 ± 99.72
β-carotene	32.4 ± 2.5	31.6 ± 4.8	16.1 ± 3.9	109.54 ± 21.19

a O: oral phase. G: gastric phase. I: intestinal phase. n.d.: not detectable.

**Figure 3:**
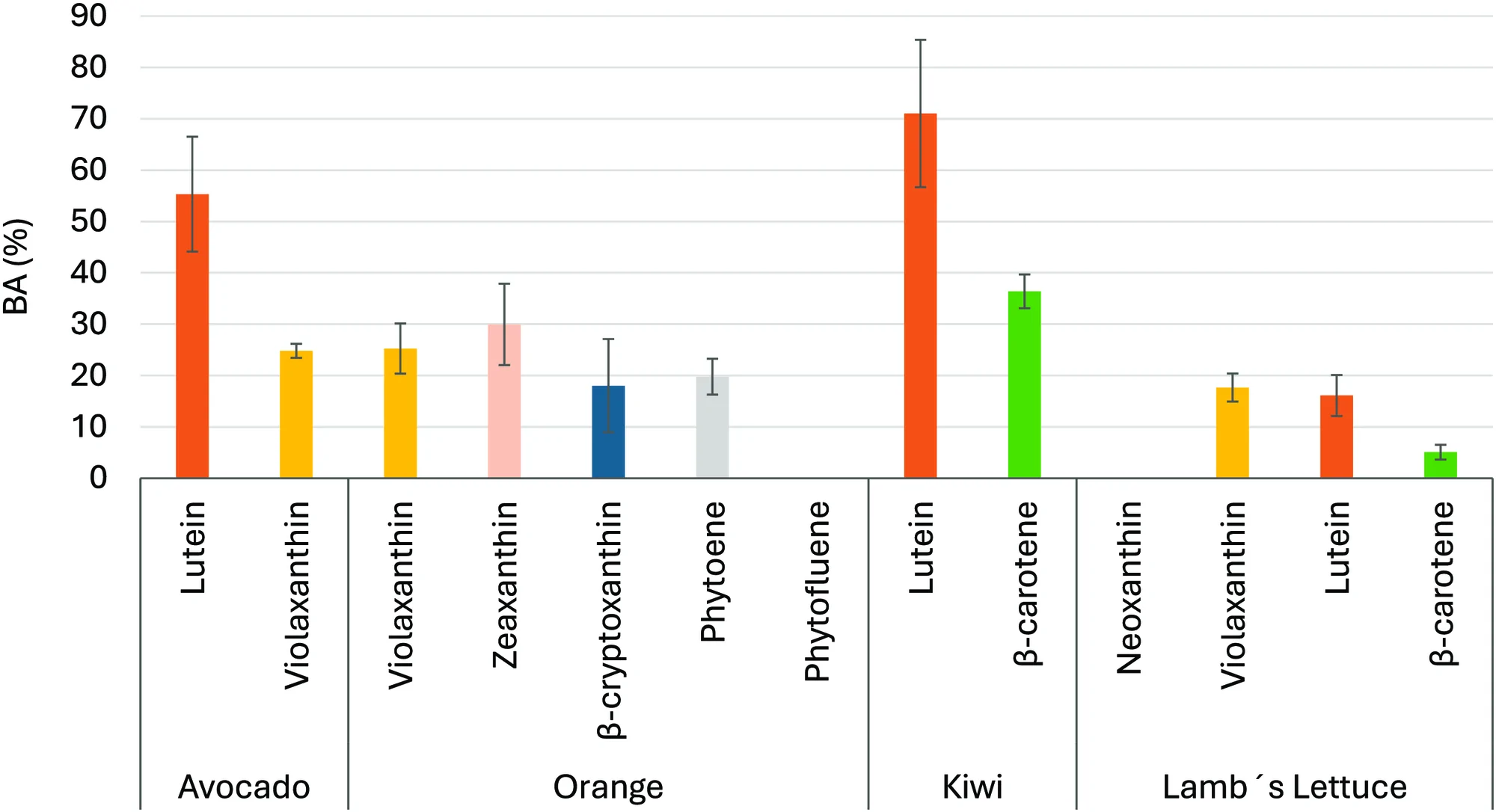
BA (%) of the major carotenoids after *in vitro* digestion in the selected pulps fruits and lamb’s lettuce.

Recovery (%) (eq 2) was calculated as the percentage of each carotenoid quantified in the digesta (noncentrifuged sample) at each digestion phase (oral, gastric, and intestinal) relative to its initial concentration in the undigested food matrix.

(carotenoidscontentaftereachdigestionphase/carotenoidscontentinundigestedfood)×100 2

This approach allows for a clearer visualization of compound stability across phases. This value is distinct from the BA calculation (eq 1), which is based on the micellar fraction. It provides an estimate of the compound’s resistance to degradation or transformation during gastrointestinal simulation. This hypothesis refers to the structural and physicochemical changes that carotenoids may undergo during digestion, such as isomerization, partial degradation, or changes in aggregation state, which can modify their release from the food matrix and their subsequent incorporation into mixed micelle. This behavior is expected considering the lipophilic nature of carotenoids and the structural characteristics of the selected food matrixes, which commonly lead to similar trends *in vitro* BA under INFOGEST conditions.

Initial findings revealed that the analyzed fruits contained higher levels of carotenoids compared to the digested ones, suggesting that these compounds were partially lost during the *in vitro* digestion. In avocado, lutein exhibits high recovery after the oral phase (83.4%) and the intestinal phase (95.8%), although it drops in the gastric phase (31.1%). Violaxanthin in avocado shows low oral recovery (32.1%). In orange, violaxanthin experiences considerable low recovery at the end of digestion (35.1%). Zeaxanthin shows a “recovery” above 100% in the oral and gastric phases, which reflects enhanced extractability during digestion rather than carotenoid BA. Mechanical disruption, enzymatic activity, and acidification likely promote the release of zeaxanthin from protein-lipid complexes and chromoplast structures. Additionally normal analytical variability at low concentrations may contribute to recoveries slightly above 100%. β-Cryptoxanthin in orange maintains moderate recovery, while phytoene shows high loss. Kiwi demonstrates good stability for both lutein and β-carotene. Lutein has consistently high recovery (around 73–80%) across all phases. β-Carotene in kiwi also shows high initial recovery (99.8%), though it decreases to 63.4% in the intestinal phase. In contrast, lamb’s lettuce exhibits low recoveries for most of its carotenoids. By the end of the intestinal phase, lutein and β-carotene showed the highest degree of degradation, retaining only 13.6% and 16.1% of their initial content, respectively. Violaxanthin recovery was also low at 19.6%.

The BA results reveal significant variability among the carotenoids and food matrixes studied (Figure 3). In avocado, lutein exhibits a notably high BA of 55.3%, indicating good availability for absorption. Violaxanthin in avocado and orange shows reasonable BA at 24.8% and 25.3%, respectively. Zeaxanthin in orange shows a BA of 30%, while β-cryptoxanthin and phytoene present intermediate values of 18.0% and 19.8%, respectively. Kiwi stands out for the high BA of lutein, reaching 71.0%, making it an excellent source of this bioavailable carotenoid. β-Carotene in kiwi also demonstrates significant BA at 36.4%. In contrast, lamb’s lettuce generally shows low BA for its carotenoids. Violaxanthin and lutein have BA of 17.7% and 16.1%, respectively. β-Carotene in lamb’s lettuce exhibits particularly low BA, with only 5.1%, implying that only a small fraction of this carotenoid is potentially absorbable.

In addition to percentage of BA, carotenoid bioaccessible content (μg/100 g) was calculated to quantify the absolute amount of carotenoid incorporated into the micellar fraction (Table 2). This parameter represents the amount of carotenoid (μg/100 g of food) that becomes potentially available for absorption and was calculated as

initialcontent×%bioaccessibility/100 3

This parameter complements the percentage of BA by providing a more realistic estimation of the carotenoid dose available for uptake.

For example, although lamb’s lettuce shows a low β-carotene BA (5.1%), its high initial concentration (2156 μg/100 g) results in a bioaccessible content of 109 μg/100 g. In contrast, foods with a high percentage of BA but low initial carotenoid levels yield much lower absolute bioaccessible amounts.

## 4 Discussion

The present study includes the *in vitro* digestion analysis of three fruits (avocado, kiwi, and orange) and lamb’s lettuce, which were used in a dietary intervention in healthy subjects to assess the effect on lutein status markers.^10^ Specifically, these foods were used because they are rich in lutein or are good contributors to lutein intake in the Spanish population.^19^ The carotenoid content in the analyzed foods aligned with reported values in scientific literature, while also exhibiting expected variations due to factors such as specific cultivar, ripeness, growing conditions, and analytical methodologies.^20^

In relation to the inclusion of cholesterol esterase in the adapted protocol, it is important to highlight that this enzyme has been widely reported to enhance the hydrolysis of xanthophyll esters under INFOGEST conditions, where pancreatic lipase alone shows limited activity toward esterified carotenoids.^9^ Although esterified carotenoids were not quantified in this study, all extracts, both from undigested foods and from digested samples, were subjected to alkaline saponification prior to chromatographic analysis. This step ensures the complete cleavage of any carotenoid esters potentially present in the matrixes and allows the quantification of carotenoids in their free form, which are the physiologically relevant species for intestinal absorption. Therefore, the analytical strategy was focused on free carotenoids after saponification rather than on profiling esterified forms or monitoring ester hydrolysis during digestion. Moreover, most of the selected foods contain carotenoids predominantly in the free form, and citrus, where esterified xanthophylls are more abundant, was included only in the preliminary adaptation step. For these reasons, cholesterol esterase was incorporated to ensure efficient ester hydrolysis during digestion, even though ester quantification was beyond the scope of the present work.

The carotenoid profile observed in the studied fruits and vegetables highlight notable compositional differences and reinforces their relevance as sources of these bioactive compounds. Hass avocado is a notable source of carotenoids such as lutein and violaxanthin. It is important to note that the concentration of these compounds can vary significantly due to factors such as variety, ripeness, growing conditions, and even the specific sampling area within the fruit.^21,22^ Postharvest handling can also lead to a decrease in certain carotenoids, with neoxanthin experiencing the most pronounced loss when stored at 20 °C.^23^ The carotenoid levels in Hass avocados are also closely linked to their total fat content, which can vary considerably.^24^

In citrus fruits such as Nave-Late oranges major identified carotenoids include β-cryptoxanthin, violaxanthin, zeaxanthin, and phytoene. Although lutein is commonly reported in oranges,^25^ it was not detected in the samples analyzed in this study. Studies on oranges consistently identify β-cryptoxanthin as a major carotenoid, though significant variation exists depending on the maturity stage and variety.^25^ For instance, while violaxanthin can be the predominant carotenoid in mature Navel and Valencia oranges, accumulating significantly during ripening phytoene and phytofluene may be present in trace amounts or not detected, indicating their minimal contribution to the carotenoid profile of these standard varieties.^26^

Hayward kiwi also demonstrated relevant concentrations of lutein and β-carotene, supporting its role as a dietary source of these carotenoids. Previous studies across orchards in central Italy found lutein to be consistently present at higher levels than β-carotene, although variation among orchards was notable.^27^ Environmental conditions, orchard microclimates, and handling practices after harvest were all considered influential factors affecting final composition.

Finally, lamb’s lettuce samples show very high concentrations of lutein and β-carotene, being a vegetable widely recognized as an excellent source of these carotenoids. While values can vary when compared to other leafy greens like spinach, where lutein is also a predominant carotenoid along with violaxanthin and all-E-neoxanthin,^28,29^ lamb’s lettuce can be considered as a carotenoid-rich leafy green.

### 4.1 Selection of Digestion Protocol and the Extraction Method for Carotenoids from Digested Foods

*In vitro* digestion models, such as the INFOGEST protocol^6,30^ are valuable tools for estimating nutrient BA. However, their application to lipophilic compounds like carotenoids presents specific challenges. Despite standardization efforts, the literature reveals that the original INFOGEST protocol is not always adequate for precisely evaluating carotenoid BA, particularly due to the nature of carotenoid esters and the critical need for efficient separation of the micellar fraction.^31,32^ For these reasons, and to adapt the assessment of carotenoid BA in the analyzed fruits and vegetables, several modifications were implemented to the standardized INFOGEST protocol, and two different protocols were tested for the *in vitro* digestion of oranges.

In both protocols, cholesterol esterase was included during the gastric phase. Although this enzyme is not part of the original INFOGEST protocol,^6,29^ its incorporation is justified because carotenoid esters naturally present in foods are typically hydrolyzed during digestion and therefore are not detected *in vivo* under usual dietary conditions. Moreover, under the conditions of the INFOGEST protocol adapted for carotenoids, ester hydrolysis is limited, which further supports the addition of cholesterol esterase to enhance the breakdown of carotenoid esters.^9,32^

Furthermore, the original INFOGEST protocol recommends using 5 g of solid food or 5 mL of liquid food as the initial sample for *in vitro* digestion. This amount is mixed with an equal volume of simulated salivary fluid (SSF), meaning a 1:1 weight/volume ratio between food and SSF during the oral phase. In protocol 2, a larger food sample (20 g) was used to achieve higher absorbance values and reduce analytical errors. This larger quantity has also been employed by other authors^32^ to evaluate carotenoid BA in tropical fruits. On the other hand, the INFOGEST protocol also does not specify exact centrifugation parameters such as speed or duration, leaving this open to adjustments depending on the sample type and the study’s objective. Centrifugation parameters, specifically speed and duration, exert a critical influence on the accurate assessment of carotenoid BA in static *in vitro* digestion models.^9^ The physical forces applied during centrifugation can impact the stability of fragile mixed micelles and lipid droplets, potentially altering their integrity and, thus, the amount of carotenoids effectively transferred into the aqueous phase.^31^ Insufficient centrifugal force or time can result in an incomplete sedimentation of solid particles and unmicellized carotenoid aggregates. This leads to a supernatant contaminated with nonbioaccessible forms, thereby artificially inflating the measured BA values. Conversely, overly aggressive centrifugation (excessively high speeds or prolonged durations) risks the unintended sedimentation of larger, yet physiologically relevant, mixed micelle structures or lipid droplets, which would consequently underestimate the true bioavailable fraction of carotenoids.^33^ For this reason, the chosen centrifugation parameters directly dictate the purity and composition of the separated supernatant, which in turn profoundly affects the efficiency and precision of subsequent carotenoid extraction and quantification, making proper adaptation crucial for reliable BA data.^31,34^ In this context, Granado-Lorencio et al.^9^ compared low-speed centrifugation with overnight static decantation at room temperature and observed that the latter provided more representative BA estimations in fruits and vegetables, particularly in matrixes with high viscosity or fiber content. They concluded that centrifugation might underestimate carotenoid recovery due to the unintended sedimentation of relevant mixed micelles and lipid droplets. In the present study, protocol 1 employed the conditions previously used in our laboratory,^12^ while protocol 2 applied the conditions that yielded the best results (20000*g* and 5 min) after conducting multiple centrifugation trials with a digested kiwi sample under this protocol to evaluate carotenoid BA in the micellar fraction. These conditions are consistent with those used by other authors and are recommended when applying the INFOGEST protocol adapted to carotenoids.^5^

It is worth mentioning that, in this study, centrifugation was employed to obtain the supernatant containing carotenoids released from the food matrix, and no filtration step was used to separate micelles specifically. Although filtration is widely used in the literature to obtain the micellar fraction *in vitro* digestion studies, several previous works have also opted for direct extraction from the supernatant without micelle isolation, particularly when dealing with complex food matrixes or when the objective is to compare relative BA across treatment.^35–37^ Our decision to omit filtration was therefore consistent with established practices in carotenoid research and was motivated by the need to preserve sample integrity for subsequent analytical procedures. While this approach may slightly reduce the precision of absolute BA values, it remains a valid and widely accepted strategy for comparative assessments of carotenoid release during digestion.

In conclusion, when applying the standardized static *in vitro* digestion model for food to assess carotenoid BA, a higher sample weight and increased centrifugation speed for separating the micellar fraction should be considered to obtain precise and improved results. The centrifugation step is particularly crucial for obtaining the fraction containing micellized carotenoids.

Because all extracts were saponified prior to chromatographic analysis, the method quantifies carotenoids exclusively in their free form and does not allow distinguishing esterified species or monitoring their hydrolysis during digestion. Therefore, the impact of cholesterol esterase cannot be directly assessed analytically. Its inclusion in the adapted INFOGEST protocol was intended to ensure efficient hydrolysis of potential xanthophyll esters during the intestinal phase, as pancreatic lipase alone shows limited activity toward these substrates. After centrifugation, carotenoid values correspond to the micellar fraction and thus represent BA rather than recovery.

### 4.2 Stability of Carotenoids during Digestion

This study examines the recovery of specific carotenoids (neoxanthin, violaxanthin, lutein, zeaxanthin, β-cryptoxanthin, phytoene, and β-carotene) in avocado, orange, kiwi, and lamb’s lettuce throughout the *in vitro* digestion phases (oral, oral + gastric and complete gastrointestinal phase), as well as their BA. The results, presented in Table 2, highlight the complex interaction between food matrix structure, carotenoid type, and digestive conditions, factors that significantly influence their fate.

In general, carotenoid recovery showed a decreasing trend as the digestion process progressed, which aligns with the inherent degradation of these compounds, known to be sensitive to factors such as pH, enzymatic activity, and oxidation during gastrointestinal transit. This pattern was clearly observed across all foods evaluated, although the extent of loss varied. The gastric phase, characterized by acidic pH and pepsin activity, is often a critical point for carotenoid stability, as previously described in the literature. The intestinal phase, with its elevated pH, presence of oxygen which, although present throughout digestion, exerts a greater impact during this phase due to the longer exposure time, and the action of bile salts and lipases, may further contribute to carotenoid degradation and isomerization.^29^ However, as shown in the study by Granado-Lorencio et al.,^38^ carotenoid stability during the salivary, gastric, and intestinal phases was quite high, exceeding 75% in most of the foods they analyzed (broccoli, spinach, sweet corn, carrot, tomato puree, red pepper, kiwi, orange, pineapple, and loquat), regardless of the type of food matrix (green or nongreen vegetable or fruit), which supports the remarkable resistance of these compounds to digestive conditions.

In avocado, lutein showed a notable fluctuation in its recovery, with a remarkable increase in its recovery up to 95.8% in the complete digestion phase. This recovery could suggest a delayed release of the carotenoid or structural changes in the food matrix that enhance its extraction and subsequent detection. Violaxanthin, on the other hand, showed a low initial oral recovery (32.1%) and was not detected in subsequent phases. It is important to consider that violaxanthin is an epoxide carotenoid with limited stability under acidic conditions. Although acid related degradation products such as 5,8 furanoid derivatives have been reported in the literature, these compounds were not specifically monitored in this study and cannot be confirmed with the analytical method used.^39^

The orange exhibited a varied behavior among its carotenoids. Zeaxanthin demonstrated notable stability, with an apparent increase during the oral + gastric phase (125.3%), later stabilizing at 84.4% in the complete gastrointestinal phase. This increase could indicate efficient release from its matrix, or enhanced analytical extraction compared to the initial sample. In contrast to previous findings in citrus (mandarin),^25^ β-cryptoxanthin stability remained relatively constant after the gastric phase (73.4% recovery) but experienced a notable decline during the intestinal phase, dropping to 50.4% of its initial content. This behavior is consistent with what has been described for its esterified forms (also in mandarin).^25^ Given the limited lipase activity during the gastric phase, it is plausible that the esters remained relatively intact during simulated digestion and were subsequently hydrolyzed during the analytical saponification process. This would favor the release of the free form, leading to greater detection in extracts from the gastric phase. Data from Granado-Lorencio et al.^38^ support this hypothesis, showing that β-cryptoxanthin esters require cholesterol esterase for effective hydrolysis, as human pancreatic lipase alone was insufficient to efficiently release the free form. However, it is also important to note that the type of esterase might have limited specificity in hydrolyzing different carotenoid esters. While the hydrolysis of xanthophyll esters (such us lutein and zeaxanthin) was over 90% in foods like pineapple, it did not reach 40% in most food matrixes, particularly low in fruits such as loquat and orange, highlighting the influence of matrix type on the release of free forms. Phytoene showed significant degradation from the beginning of the digestive process, with limited recovery in the oral phase (29%) and losses around 70% across all phases. This early degradation may be attributed to the low chemical stability of phytoene under oxidative and enzymatic conditions, as well as its limited structural protection within the orange matrix. Unlike more conjugated carotenoids, phytoene has fewer conjugated double bonds, making it more susceptible to oxidation in the presence of oxygen and to digestive enzymes.^40,41^ Additionally, its low affinity for lipid structures could hinder its incorporation into mixed micelles, reducing its protection against intestinal degradation.^42^ Violaxanthin, as observed in avocado, suffered significant loss from the oral to intestinal phases. In line with this, Granado-Lorencio et al.^9^ described a notable decrease in epoxidized carotenoids during the gastric phase, which was particularly critical for compounds such as violaxanthin and neoxanthin. This degradation was attributed to acid hydrolysis and epoxide group rearrangement, leading to new derivatives that may not be detected through conventional chromatographic methods.

In kiwi, lutein exhibited high stability throughout the *in vitro* digestive process, with recoveries of 80.3% in the oral phase, 78.3% in the oral + gastric phase, and 73.7% in the complete gastrointestinal phase. This remarkable stability may be attributed to kiwi’s cellular structure, which is rich in bioactive compounds and relatively soft, facilitating carotenoid release, as well as the presence of actinidin, a proteolytic enzyme that may aid in the disruption of the protein matrix.^43^ Lutein stability during digestion is enhanced in matrixes rich in bioactive compounds, particularly antioxidants such as vitamin C, phenolics, and other reducing agents naturally present in fruits like kiwi and avocado. These constituents can scavenge reactive oxygen species generated during gastric and intestinal digestion, thereby protecting the polyene chain of lutein from oxidative cleavage. In addition, bioactive-rich matrixes often contain polar lipids and natural emulsifiers that promote the formation of stable colloidal structures, which physically shield lutein from isomerization and oxidation. This combined antioxidant and structural protection mechanistically explains the higher lutein stability observed in these matrixes.^17^ Meanwhile, β-carotene also showed excellent initial recovery (99.8% in the oral phase), followed by a moderate loss in the complete gastrointestinal phase, suggesting some susceptibility to degradation. This high initial recovery could be linked to kiwi’s soft cellular structure and low insoluble fiber content, which facilitates mechanical disruption and carotenoid release during the oral phase. However, the moderate loss in the complete gastrointestinal phase suggests some susceptibility of β-carotene to degradation under more aggressive physiological conditions, such as the presence of bile salts and pancreatic enzymes. These results are consistent with those described by Granado-Lorencio et al.,^9^ except for the β-carotene loss in the intestinal phase. They observed over 80% stability for lutein and over 90% for β-carotene in kiwi during the salivary, gastric, and intestinal phases, confirming their resistance to simulated digestive conditions.

Lamb’s lettuce exhibited the lowest overall stability for all detected carotenoids. Lutein showed very low recovery (27.0% oral, 12.1% oral + gastric and 13.6% complete gastrointestinal phase). Similarly, violaxanthin (20.1% complete gastrointestinal phase) and β-carotene (16.1% complete gastrointestinal phase) demonstrated significant reductions. Studies conducted on other leafy green vegetables, such as spinach, have also shown high loss values for these carotenoids when their fresh forms were subjected to digestion. In this regard, lutein, β-carotene, and violaxanthin exhibited, respectively, 24%, 60%, and 95% losses in the study by Courraud et al.,^44^ and losses of 76% (lutein) and 50% (β-carotene) in the work by Rich et al.^45^ Granado-Lorencio et al.^38^ described that lettuce showed the lowest carotenoid stability during *in vitro* digestion, with losses exceeding 25% in all phases, which aligns with the low values observed in lamb’s lettuce. As for neoxanthin, it was detected only in the oral phase of the *in vitro* digestion process, while it was not observed in the oral + gastric or complete gastrointestinal phases. This behavior can be attributed to its high structural sensitivity to aggressive digestive conditions. In particular, the acidic environment of the gastric phase favors the opening of the epoxide ring and the transformation of neoxanthin into derivatives like neochromes, which are not detectable by conventional chromatographic analysis methods.^46,47^ Additionally, the action of pancreatic enzymes and bile salts in the intestinal phase can promote further degradation or hinder its efficient incorporation into micelles, complicating its subsequent recovery.^48^ In contrast, during the oral phase, characterized by a neutral pH and the absence of intensive digestive enzymes, neoxanthin remains stable, allowing for its detection.

In general terms, lutein (in avocado and kiwi) and β-carotene (in kiwi) are more stable in fruits than in lamb’s lettuce, whereas violaxanthin exhibits very low stability in both types of food.

### 4.3 Carotenoids BA

The findings in the present work demonstrate a generally higher BA for lutein in fruits (avocado, orange, and kiwi) compared to leafy green vegetables (lamb’s lettuce), which is consistent with existing literature.^49^ While the carotenoid content in vegetables is typically higher than in fruits, especially for lutein, β-carotene, neoxanthin, and violaxanthin, numerous studies have reported better carotenoid transfer to micelles from fruits than from vegetables. Xanthophylls (lutein, zeaxanthin, and β-cryptoxanthin) are often highly bioaccessible in fruits (ranging from 50% to 100%), possibly due to their localization in chromoplasts dissolved within oil droplets, which may facilitate their release.^49^ This pattern was confirmed by Granado-Lorencio et al.,^38^ who observed that green vegetables such as spinach, lettuce, and broccoli exhibited lower transfer rates of lutein and β-carotene to the micellar phase compared to fruits like kiwi, orange, and loquat. These differences were attributed to the intracellular localization of carotenoids (e.g., in chloroplasts) and the presence of dietary fibers that limit their release. Accordingly, positive correlations were identified between the fat content of foods and the BA of carotenoids like lutein and β-carotene, while negative correlations with fiber content. Although these differences were not statistically significant in all cases, they suggest that lipid composition may favor the incorporation of carotenoids into mixed micelles.

In avocado, the BA values for lutein and violaxanthin are notably high (55.3% and 24.8%, respectively). As an oleaginous fruit, avocado contains lipid-soluble carotenoids embedded in a lipid-rich matrix composed primarily of monounsaturated fatty acids and phospholipids,^50^ which facilitates their dispersion and subsequent micellization during digestion, a critical step for intestinal absorption. However, it should be noted that the fruit ripening stage affects the bioavailability of fat-soluble carotenoids from avocado; therefore, these values may vary depending on the maturity stage of the analyzed sample.^51^

Although kiwi is not a lipid-rich fruit like avocado, its matrix structure may play a crucial role in carotenoid BA. The specific composition of the cell wall and the organization of chromoplasts where carotenoids are stored in kiwi could permit efficient release, as the progressive solubilization of pectic polysaccharides and loss of galactose during ripening loosens the cell wall matrix, while the differentiation of chromoplasts into carotenoid-rich crystalline or globular structures enhances BA).^52^ Furthermore, lutein in fruits is often found in free or esterified forms with fatty acids, and the latter might be more bioavailable than unesterified forms, depending on the enzymatic capacity for hydrolysis in the intestine. In the study by Granado-Lorencio et al.,^38^ lutein in kiwi exhibited a 59% incorporation into the micellar phase, while β-carotene reached 35%, confirming their relatively high BA compared to other fruits evaluated. In the present study, lutein BA was considerably higher (71.0%) than the values reported for other nonfatty fruits, in which intestinal BA of lutein typically remains below 50%.^38^ Regarding β-carotene, BA was lower than that observed for lutein (36.4%). This value aligns with previous studies such as Mapelli-Brahm et al.,^40^ who reported intestinal BA of β-carotene below 50% in nonfatty fruits such as mango and papaya, and highlighted that food matrix and processing degree significantly influence carotenoid stability. Similarly, Mapelli-Brahm et al.^41^ reported that β-carotene, despite being susceptible to oxidation and isomerization during intestinal digestion, may experience reduced micellar incorporation because of these transformations. Within this context, kiwi stands out as an efficient source of β-carotene, demonstrating near-complete initial release and moderate intestinal degradation, making it a promising fruit to enhance intake of lipophilic carotenoids in low-fat diets.

On the other hand, orange exhibits more variable behavior. Violaxanthin (25.3%), zeaxanthin (30.0%), β-cryptoxanthin (18.0%), and phytoene (19.8%) display generally low BA values. These values may be related to the potential degradation of more labile carotenoids (such as epoxides), or to interactions with matrix components that sequester them as digestion progresses. The presence of pectins and other nonstarch polysaccharides in orange may form a network that, while facilitating initial release, could hinder complete micellization of certain carotenoids during the final phases.^53^ In this regard, Granado-Lorencio et al.^38^ observed that the hydrolysis of xanthophyll esters like β-cryptoxanthin in orange was incomplete (<40%), which limits the availability of the free form for micellar incorporation. Indeed, consistent with our results, these authors described micellar BA below 40% for this carotenoid. Phytoene stands out for its relatively high BA, frequently reported as having comparable or even higher bioaccessibilities than xanthophylls. The value obtained in the present study (19.8%) aligns with those reported for mandarin cv. Clementine (*C. clementina*) (20–30%)^52^ and mandarin cvs “Ponkan”, “Rio”, and “Murcott” (22–32%).^32^ In contrast, studies conducted on more processed matrixes such as tomato or apricot have described significantly higher phytoene BA (up to 97%), suggesting that the intact cellular structure of whole orange may limit both its release and stability.^40^ These results reinforce the need to consider the food matrix and digestive environment when evaluating the BA of linear carotenoids such as phytoene. The study by Granado-Lorencio et al.^38^ also showed that phytoene BA in fruits like orange was below 20%, further emphasizing the influence of the food matrix on micellization efficiency.

For lamb’s lettuce the BA of both lutein and β-carotene showed low values (16.1% and 5.1%, respectively). This aligns with previously reported data, which demonstrate that, compared to fruits, dark green leafy vegetables tend to exhibit significantly lower carotenoid BA (typically ranging between 5–10%). This phenomenon may be attributed to the strong association of carotenoids with chloroplast protein structures, which are encapsulated by dense cell walls rich in fiber, acting as a physical barrier that limits carotenoid release into the intestinal lumen during digestion. Furthermore, the low lipid content in these vegetables may also reduce micellization efficiency.^38,49,54^ In addition, the presence of dietary fiber, pectin, and mucilaginous compounds can increase chyme viscosity, thereby decreasing the diffusion of liberated carotenoids and impeding micelle formation, which further limits their BA. For this reason, processing techniques such as steaming or oil addition can significantly improve this BA.^55,56^

The inclusion of bioaccessible content values also provides a more nuanced interpretation of the nutritional relevance of the studied foods. While percentage of BA reflects the efficiency of micellarization, it does not capture the actual amount of carotenoid available for absorption. By integrating both the initial carotenoid concentration and its micellarization efficiency, the bioaccessible content highlights important contrasts among matrixes. For example, lamb’s lettuce, despite its low BA percentages, delivers substantially higher absolute amounts of micellarized lutein and β-carotene than fruits with higher BA but much lower carotenoid density. This reinforces that foods with modest BA values may still represent nutritionally meaningful sources when their intrinsic carotenoid content is high. Therefore, interpreting BA together with bioaccessible content offers a more realistic assessment of the potential contribution of each food to carotenoid intake and physiological status.

In summary, the BA findings found in the present study demonstrate that lutein is more bioaccessible than β-carotene in both lamb’s lettuce (16.1% vs 5.1%) and kiwi (71.0% vs 36.4%). This aligns with previous studies reporting higher BA for xanthophylls (lutein, zeaxanthin, violaxanthin, neoxanthin) compared to carotenes (β-carotene).^49,57^ Furthermore, the high BA of lutein in avocado and kiwi is consistent with the hypothesis that lipids (in avocado) and matrix structure (in kiwi) facilitate its release. Other investigations into lutein bioavailability from various plant sources confirm that dietary sources differ in their capacity to elevate plasma lutein levels, a variability attributed to differences in BA based on the specific food matrix.^38,58^

The differences observed in carotenoid BA across matrixes can be explained by their compositional characteristics. Lipid-rich matrixes such as avocado facilitate carotenoid solubilization and micellar incorporation by promoting efficient lipid digestion and mixed micelle formation, which is consistent with the high BA values observed for lutein and violaxanthin. In contrast, matrixes with low lipid content and high levels of insoluble fiber, such as lamb’s lettuce, hinder carotenoid release by retaining pigments within rigid cell walls and chloroplast–protein complexes, thereby reducing micellarization efficiency. The presence of pectins and other soluble fibers in fruits like orange can increase digesta viscosity and limit the diffusion of carotenoids into mixed micelles, contributing to their moderate BA. Additionally, divalent cations such as Ca^2+^ and Mg^2+^, abundant in leafy greens, can interact with pectins and cell wall polysaccharides to form cross-linked networks that further restrict carotenoid liberation and emulsification. These matrix-dependent factors collectively explain the variability in BA observed among the studied foods.

This study presents several strengths, including the use of a modified INFOGEST protocol tailored to carotenoid-rich matrixes, the inclusion of multiple food types relevant to the Spanish diet, and the parallel assessment of both carotenoid stability and BA. However, certain limitations should be acknowledged. First, enzymatic activities were not experimentally verified but rather assumed based on supplier specifications, which may introduce variability in digestion efficiency. Second, although cholesterol esterase was included to enhance xanthophyll ester hydrolysis, no parallel digestion was performed without it, limiting our ability to isolate its specific contribution. Third, the comparative evaluation of digestion protocols was conducted exclusively using orange as the test matrix, and the study did not include formal statistical comparisons between all matrixes, which may affect the generalizability of the selected conditions. Additionally, although centrifugation conditions were adapted based on analytical performance, we acknowledge that microscopic characterization of digesta colloidal structures could provide complementary insight into micelle formation and phase separation. This approach may be incorporated in future studies to further elucidate the physical effects of centrifugation on mixed micelles. Lastly, and most significantly, the use of a static *in vitro* digestion model means that our values, while highly informative, cannot fully replicate the complexity of human absorption and metabolism (bioavailability). Therefore, the reported BA results should be interpreted as predictive estimates that require confirmation through *in vivo* human trials.

## †

Present address: Faculty of Health Sciences, Universidad Francisco de Vitoria, Carretera de Pozuelo a Majadahonda, KM1.800, 28223 Madrid, Spain
